# Molecular identification of *Plasmodium* spp. and blood meal sources of anophelines in environmental reserves on São Luís Island, state of Maranhão, Brazil

**DOI:** 10.1186/s13071-017-2133-5

**Published:** 2017-04-26

**Authors:** Mayra Araguaia Pereira Figueiredo, Silvia Maria Di Santi, Wilson Gómez Manrique, Luiz Ricardo Gonçalves, Marcos Rogério André, Rosangela Zacarias Machado

**Affiliations:** 1 Immunoparasitology Laboratory, School of Agrarian and Veterinary Sciences (FCAV), Universidade Estadual Paulista (UNESP) Jaboticabal Campus, Jaboticabal, SP Brazil; 2 Center for Malaria Studies, Superintendence of Control of Endemic Diseases, State Secretariat of Health of São Paulo/Institute of Tropical Medicine of São Paulo (IMT-SP), University of São Paulo (USP), São Paulo, SP Brazil; 3 Veterinary Pathology Laboratory, Brazil University, Descalvado Campus, Descalvado, SP Brazil

**Keywords:** *Anopheles*, Feeding habit, PCR, Malaria, Vector

## Abstract

**Background:**

Considering the diversity of feeding habits that females of some species of anophelines present, it is important to understand which vertebrates are part of blood food sources and how important is the role of each in the ecoepidemiology of malaria. There are many vector species for *Plasmodium* spp. in the State of Maranhão, Brazil. In São Luís Island, *Anopheles aquasalis* is the main vector for human malaria; this species is abundant in areas with primates that are positive for *Plasmodium. Anopheles aquasalis* has natural exophilic and zoophilic feeding behavior, but in cases of high density and absence of animals, presents quite varied behavior, and feeds on human blood. In this context, the objective of the present study was to identify *Plasmodium* spp. and the blood meal sources of anophelines in two environmental reserves on São Luís Island, state of Maranhão, using molecular methods.

**Methods:**

Between June and July 2013, female anophelines were collected in the Sítio Aguahy Private Reserve, in the municipality of São José de Ribamar, and in the Sítio Mangalho Reserve, located within the Maracanã Environmental Protection Area, in the municipality of São Luís. CDC-type light traps, Shannon traps and protected human bait were used during three consecutive hours in peridomestic and wooded areas. Pools of anophelines were formed using mosquitoes of the same species that had been caught at the same site on the same date. A genus-specific amplification protocol based on the 18S rRNA gene was used for qPCR and cPCR.

**Results:**

A total of 416 anophelines were collected, of the following species: *An. aquasalis* (399), *An. mediopunctatus* (3), *An. shannoni* (1), *An. nuneztovari* (*sensu lato*) (1), *An. goeldii* (1), *An. evansae* (2) and *An.* (*Nyssorhynchus*) sp. (9), comprising 54 pools. Two pools were positive for *Plasmodium* (2/54) based on the 18S rRNA gene. In the phylogenetic analysis using the maximum likelihood method, based on a 240 bp fragment of the 18S rRNA gene, it was found that the sequences of *Plasmodium* sp. amplified from pools of *An. aquasalis* (pool 2) and *An. nuneztovari* (*s.l*.) (pool 10) were phylogenetically related to a clade of *P. falciparum* isolates from India, and to a clade of *Plasmodium* sp. isolates from psittacines in Brazil, respectively. Cat, dog and human DNA were identified in the blood meals of the anophelines sampled.

**Conclusion:**

The species *An. aquasalis* was the most abundant anopheline species in São Luís Island. *Plasmodium* spp. DNA was detected, thus confirming the importance of this species as the main vector on São Luís Island, Brazil. In addition, the presence of *An. nuneztovari* (*s.l*.) with DNA positive for *Plasmodium* spp. confirms its importance as a secondary vector.

## Background

In the Americas, Brazil is the country that presents the highest number of cases of malaria (42%), with a predominance of *Plasmodium vivax* [1]*.* In this country, a complex epidemiological situation is observed, with areas without transmission, areas with low transmission and areas with high transmission of the disease [1]. Malaria cases are concentrated in the states that comprise the Legal Amazon Region (Acre, Amazonas, Rondônia, Roraima, Amapá, Pará, Maranhão, Mato Grosso and Tocantins), that accounts for 99.6% of the cases [2]. Among these states, Maranhão has registered the lowest number of notifications of the disease, presenting a 61% reduction in the number of cases between 2014 (1,327 cases) and 2015 (517 cases) and, consequently, the lowest number of deaths [2]. The parasite species responsible for the highest number of cases in Maranhão is *P. vivax*, and the anopheline species that are considered to be the main vectors are *Anopheles aquasalis* on the coast and *An. darlingi* in the interior of this state [3].

São Luís Island, where the capital of the state of Maranhão is located, is composed of the municipalities of São Luís, São José de Ribamar, Paço do Lumiar and Raposa. *Anopheles aquasalis* is the main vector for malaria on this island*.* However, several secondary vector species have now been identified, including *An. evansae*, *An. galvaoi*, *An. albitarsis*, *An. nuneztovari* and *An. triannulatus davisi* [4]*.* Nevertheless, there are few reports on *Plasmodium* sp. or the feeding habits of anophelines in the state of Maranhão [5].

Acrodendrophic anopheline species are important in the evaluation of maintenance of simian malaria and transmission to humans. Some species are particularly studied for their insertion in the ecoepidemiology of human malaria and its presence in areas of malaria-positive Neotropical primates [6]. Knowedge of the basic ecology of the feeding habits of main and secondary vectors of malaria in forest environments provides relevant information on the parasite-host-vector relationships. This makes it possible to determine the potential reservoirs and propose more effective strategies for disease control. It is known that in communities in which the main malaria vector is a mosquito that is not strictly anthropophilic, the prevalence of the disease is lower [7].

The objective of the present study was to identify *Plasmodium* spp. and feeding sources in anophelines collected in two environmental reserves on São Luís Island, the State of Maranhão, Brazil.

## Methods

### Research areas

**Fig. 1:**
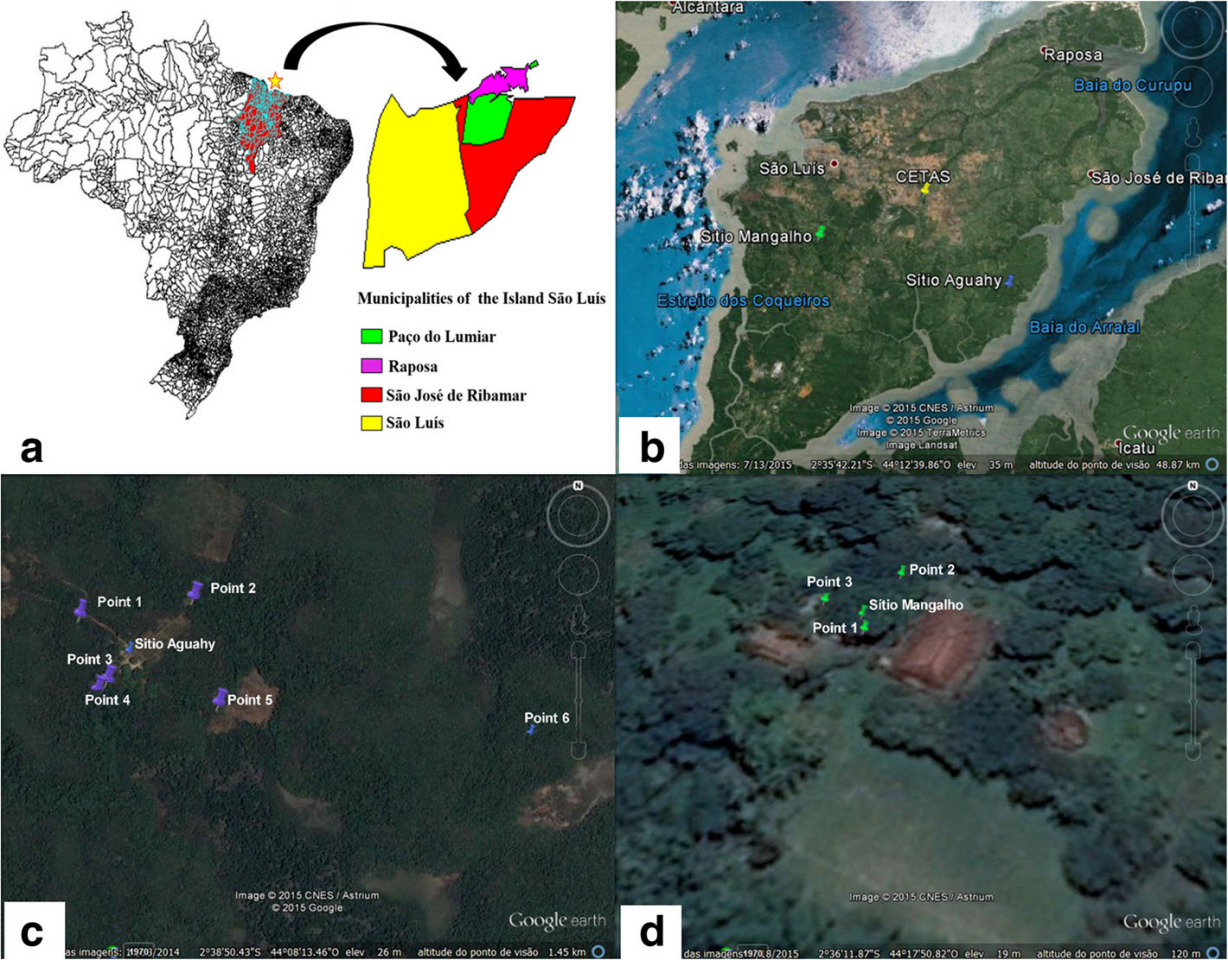
Study areas. **a** Locations of the municipalities of São Luís Island (star), in the state of Maranhão, Brazil (MapInfo™ Professional 7.5 SCP). **b** Map of São Luís Island showing the locations of the two reserves (Sítio Aguahy and Sítio Mangalho, Maracanã EPA). **c** Mosquito sampling points in the Sítio Aguahy Reserve (São José de Ribamar), point 1 (2°38'42.13"S, 44°08'33.07" W), point 2 (2°38'40.67"S, 44°08'23.62"W), point 3 (2°38'48.21"S, 44°08'31.98"W), point 4 (2°38'48.58"S, 44°08'32.23"W), point 5 (2°38'49.85"S, 44°08'21.76"W), point 6 (2°38'52.49"S, 44°07'54.40"W). **d** Mosquito sampling points in Sítio Mangalho, Maracanã EPA (São Luís), point 1 (2°36'11.05"S, 44°17'51.35"W), point 2 (2°36'10.58"S, 44°17'50.96"W), point 3 (2°36'10.84"S, 44°17'51.57"W) (Google Earth®)

São Luís Island (Fig. 1) is located in the northern of the state of Maranhão (northeastern Brazil) and is divided into four municipalities, which include the state capital, São Luís. The climate is tropical, with relative air humidity above 80% and high temperatures (approximately 26 °C) throughout the year [8]. Anophelines were caught in two environmental reserves located in the rural zones of two municipalities on the island (São José de Ribamar and São Luís): (i) Sítio Aguahy Private Reserve (2°38'6"S, 44°08'2"W), in the municipality of São José de Ribamar, has an area of 600 ha, composed of mangrove swamps (120 ha), sandspit (*resting*) vegetation (50 ha) and fragments of Amazon Forest vegetation. Although this is one of the best-preserved areas on São Luís Island, it is under intensive human action because of six communities living in the areas surrounding of the reserve and which use the reserve for recreation and fishing; (ii) Sítio Mangalho (2°36'11.31"S, 44°17'50.82"W) is a private property of approximately 11 ha [9], located within the Maracanã Environmental Protection Area (EPA), in the municipality of São Luís. The soil of EPA of Maracanã is chemically poor, and the vegetation, like the Amazon Forest, is maintained by organic matter from the soil produced by the flora. Regarding hydrography, the Maracanã River stands out as the main surface water resource [10]. All sampling points were registered using a global positioning system (GPS) device (Garmin eTrex Vista®).

### Mosquito sampling and identification

The adult anophelines were collected during the rainy season, between June and July 2013, using CDC-type light traps [11], Shannon traps (with light bait) [6, 12], and protected human bait (using Castro aspirators). Shannon traps consist of a white cotton fabric structure with a light source in it, tied at 20 cm from the ground [6]. The protected human bait method consists of the use of properly trained people as a thermal, olfactory and visual attraction for mosquitoes [13]. Before landing on the individual, the mosquito is captured by Castro aspirators [13, 14]. Mosquitoes were caught on five or six consecutive days, over a 4-h period (5:30 pm to 9:30 pm), at three points in Sítio Mangalho (23–27 June and 16–31 July 2013) and six in the Sítio Aguahy Reserve (5–22 June and 5–10 July 2013), in peridomestic and forested areas (near to water collection sites and primate movement routes).

The adult mosquitoes were stored alive in plastic containers (12 × 10 × 17 cm) with nylon screen nets and labelled according to sampling time and location. They were then sent to the Entomology Laboratory of the National Healthcare Foundation (FUNASA), at its main office in São Luís, for individual morphological identification using the identification key proposed by Consoli & Lourenço-de-Oliveira [6]. Mosquitoes were then separated into pools containing up to 10 individuals of the same species that were caught at the same location, in the same type of trap, in the same day and at the same time, and pools placed in 1.5 ml microtubes with isopropyl alcohol (P.A., ACS reagent). When there was only one specimen of a given species, this was stored individually.

### Extraction of genomic DNA from *Plasmodium* spp. and DNA amplification using conventional PCR

DNA extraction from anophelines was performed in pools. Each mosquito was cut into several segments using a sterilised scalpel blade. DNA was then extracted using QIAamp DNA Mini-Kit (Qiagen®, Valencia, California, USA), following the manufacturer’s recommendations. The extracted DNA was eluted in a final volume of 50 μl, and its concentration was quantified using a spectrophotometer (Nanodrop, Thermo Scientific®, Dubuque, Illinois, USA), by measuring the absorbance. Finally, the DNA samples were stored at -20 °C.

The cPCR protocol, based on the 18S rRNA gene, was performed using the primers and thermal sequences described by Santos et al. [15] (Table 1). In summary, the first reaction, with a final volume of 50 μl, contained: 1× *Taq* buffer (10 mM of Tris-HCl at pH 8.3 and 50 mM of KCl), 4 mM of MgCl_2_ (Life Technologies®, Carlsbad, California, USA), 0.8 mM of deoxynucleotide triphosphates (Life Technologies®), 0.25 μM of the primers rPLU1 and rPLU6R, 1.25 U of Platinum® *Taq* DNA Polymerase (Life Technologies®), and 5 μl of DNA. The volume was completed with ultrapure sterilised and autoclaved water (Life Technologies®). The second reaction, with a final volume of 20 μl, consisted of: 1× *Taq* buffer (10 mM of Tris-HCl at pH 8.3 and 50 mM of KCl), 4 mM of MgCl_2_, 0.4 mM of deoxynucleotide triphosphates, 0.25 μM of the primers rPLU3 and rPLU4, 0.5 U of Platinum *Taq* DNA Polymerase (Life Technologies®) and 2 μl of the amplified product from the first reaction. The volume was again completed with ultrapure sterilised and autoclaved water (Life Technologies®). The 240 bp products were observed using electrophoresis on 1% to 2% agarose gel under UV light and were purified using the Silica Bead DNA Gel Extraction Kit (Thermo Scientific®, São Paulo, Brazil).

**Table 1:** Description of the primers and amplification conditions of the protocol by Santos et al. [15], performed for molecular detection of *Plasmodium* spp. based on 18S rRNA gene

Primer	Primer sequence/amplification conditions	Product size (bp)
rPLU6R	5'-CGTTTTAACTGCAACAATTTTAA-3'	600
rPLU1	5'-TCAAAGATTAAGCCATGCAAGTGA 3'	
1st reaction	95 °C for 5 min; 25 cycles of 95 °C for 1 min, 58 °C for 2 min, 72 °C for 2 min; and a final extension step at 72 °C for 5 min	
rPLU3 rPLU4	5'-TTTTTATAAGGATAACTACGGAAAAGCTGT-3' 5'-TACCCGTCATAGCCATGTTAGGCCAATACC-3'	240
2nd reaction	95 °C for 5 min; 30 cycles of 95 °C for 1 min, 64 °C for 2 min and 72 °C for 2 min; and a final extension at 72 °C for 5 min	

### Phylogenetic analysis

Phylogenetic reconstructions were performed based on a 240 bp fragment of the 18S rRNA gene, sequenced in both directions using the ABI 3730 DNA Analyzer (Life Technologies®, Applied Biosystems). Consensus sequences were obtained using the CAP3 software (http://doua.prabi.fr/software/cap3). The nucleotide sequences produced from each sample were compared with the sequences deposited in the GenBank database, using BLASTn to analyze similarities [16]. Sequence alignment was performed through ClustalW [17], and was manually adjusted using Bioedit v. 7.0.5.3 (Carlsbad, CA, USA) [18]. Phylogenetic inferences based on the maximum likelihood criterion were made using RAxML-HPC BlackBox 7.6.3 [19] through the CIPRES Science Gateway [20]. The Akaike information criterion was applied using Mega 6.06 to identify the most appropriate nucleotide substitution model [21]. The GTR + G model was chosen and applied in the maximum likelihood of the 18S rRNA alignment.

### Real-time PCR assay (qPCR) to detect *Plasmodium* spp

A genus-specific amplification protocol based on the 18S rRNA gene was used, as described by Lima et al. [22], with modification of the DNA volume for greater sensitivity. The reaction was standardized for a final volume of 25 μl, containing 0.5 μM of the primers M60 and M61, 0.3 μM of the hydrolysis probe M62, 12.5 μl of 1× TaqMan Universal Master Mix (Life Technologies®), 4.25 μl of ultrapure water and 5 μl of DNA (Table 2). PCR amplifications were conducted in Low-Profile Multiplate™ Unskirted PCR Plates (BioRad, Hercules, CA, USA) using CFX96 Thermal Cycler (BioRad). The thermal amplification conditions are described in Table 2.

**Table 2:** Description of the primers, probe and amplification conditions of the qPCR protocol by Lima et al. [22], performed for molecular detection of *Plasmodium* spp. based on 18S rRNA gene

Primer/probe	Primer sequence/amplification conditions	Product size (bp)
M60	5'-ACA TGG CTA TGA CGG GTA ACG-3'	84
M61	5'-TGC CTT CCT ATG TAG TGG TAG CTA-3'	
M62	5'-FAM TCA GGC TCC CTC TCC GGA ATC GA-TAMRA-3'	
Reaction	50 °C for 2 min and 95 °C for 10 min; 40 cycles of 94 °C for 30 s and 60 °C for 1 min	

The sensitivity of the qPCR reactions was determined using pGEM-Teasy plasmid serial dilutions (Promega®, Madison, Wisconsin, United States) (10^5^, 10^4^, 10^3^, 10^2^, 10^1^ and 10^0^) with an insert of 84 bp in TE buffer (10 mmol/l of Tris-HCl and 0.1 mmol/l of EDTA; at pH 8.0). The number of copies of plasmids was determined according to the following formula: (Xg/μl DNA/[plasmid length in bp × 660]) × 6.022 × 10^23^ × plasmid copies/μl.

The amplification efficiency (E) was calculated from the slope of the standard curve in each assay using the following formula: (E = 10-1/slope). To determine the detection limit of the TaqMan assay, the standard curves produced through 10-fold dilutions were used to determine the amount of DNA that can be detected with 95% sensitivity [23].

The qPCR assays followed the descriptions of the Minimum Information for Publication of Quantitative Real-time PCR Experiments (MIQE) [23].

### Construction of standard plasmids for use in the qPCR reaction

Positive controls of *P. falciparum* and *P. malariae* DNA were used in the amplification reactions of the cPCR to produce the insert for cloning, with the same pair of primers as used in the qPCR reaction [22]. Briefly, the total volume of the reaction was 50 μl, with 4 μl of DNA, 32.75 μl of sterilized ultrapure water, 1× *Taq* buffer (10 mM of Tris-HCl at pH 8.3 and 50 mM of KCl), 0.8 mM of deoxynucleotide triphosphates, 2 mM of MgCl_2_, 0.2 μM of the primers M60 and M61, and 1.25 U of Platinum *Taq* (Life Technologies®). The reaction started with a cycle at 95 °C for 10 min, followed by 32 amplification cycles of 94 °C for 30 s, 60 °C for 30 s and 72 °C for 30 s.

The 84 bp fragments were observed on 2% agarose gel. They were then purified using the Silica Bead DNA Gel Extraction Kit (Thermo Scientific®, São Paulo, Brazil) and were cloned using the pGEM-T Easy Vector System (Promega®), following the manufacturer’s recommendation. The binding products were transformed into *Escherichia coli* One Shot Match 1TM Chemically Competent Cells (Life Technologies®). Plasmid DNA was extracted using the QIAprep Miniprep Kit (Qiagen®).

### Identification of the mosquito blood meal sources through PCR

PCR amplifications with the aim of analysing the anopheline blood meal sources were performed as described by Chang et al. [24]. PCR was based on the amplification of sequences of the mitochondrial cytochrome *b* gene from humans, birds, cats and domestic dogs, using the primers previously described by these authors (Table 3). To identify the DNA from Neotropical primates, the primers described by Canavez et al. [25] were used, based on the intron-2 region of the β_2_-microglobulin gene: F7 (5′-CTC ACC ACC CAA GAC AGT AAA GT-3′) and R6 (5′-TGA AAA AGA CGA TGG AGA AAG AAA A-3′), producing a 812 bp fragment. Briefly, the reaction had a final volume of 25 μl: 5 μl of DNA, 25 μM of each primer, 2.5 mM of each dNTP, 1.5 mM of MgCl_2_ and 0.7 U of Platinum *Taq* DNA Polymerase. The thermal conditions of the amplification were: 95 °C for 30 s, followed by 30 cycles of 95 °C for 20 s, 60 °C for 30 s and 72 °C for 30 s and a final extension at 72 °C for 5 min.

**Table 3:** Sequences of primers based on the cytochrome *b* (*cytb*) gene that were used to identify the food sources of anopheline mosquitos [24]

Food source tested	Primer sequence	Product size (bp)
Domestic cat	TTCTCAGGATATACCCTTGACA	180
	GAAAGAGCCCATTGAGGAAATC	
Human	TTCGGCGCATGAGCTGGAGTCC	228
	GTRTARTAGGGRTGRAATC	
Domestic dog	GAACTAGGTCAGCCCGGTACTT	153
	CGGAGCACCAATTATTAACGGC	
Birds	GACTGTGACAAAATCCCNTTCCA	508
	GTCTTCATCTYHGGYTTACAAGAC	
Reaction	94 °C for 2 min; 35 cycles of 94 °C for 30 s, 54–70 °C^a^ for 30 s, 72 °C for 30 s; and a final extension step at 72 °C for 20 min	

^a^Depending on the annealing temperature of each oligonucleotide (see Chang et al. [24])

## Results

### Identification and distribution of anophelines

A total of 407 (97.8%) mosquitoes of six different species were caught between June, and July 2013 were successfully identified (Table 4). Some specimens (*n* = 9) could not be identified down to the species level due to scale loss, and thus were classified as *Anopheles* (*Nyssorhynchus*) sp. (2.2%). All collected specimens were identified as belonging to the subgenera *Nyssorhynchus* and *Anopheles*.

**Table 4:** Number of specimens of *Anopheles* spp., according to subgenus, species, number of pools and locations where they were caught on São Luís Island, state of Maranhão, in 2013

Subgenus	Species	No. of specimens	No. of pools	Location
*Nyssorhynchus*	*An. aquasalis*	399	44	Sítio Aguahy (*n* = 366) and Sítio Mangalho (*n* = 33)
	*An. goeldii*	1	1	Sítio Mangalho
	*An. evansae*	2	1	Sítio Mangalho
	*An. nuneztovari* (*s.l.*)	1	1	Sítio Aguahy
	*Anopheles* sp.	9	3	Sítio Aguahy (*n* = 8) and Sítio Mangalho (*n* = 1)
*Anopheles*	*An. shannoni*	1	1	Sítio Aguahy
	*Annn mediopunctatus*	3	3	Sítio Aguahy

In Sítio Aguahy (municipality of São José de Ribamar), a total of 379 female anophelines were caught at six sampling points: one mosquito was caught in the external area of a house (veranda), and the remaining 378 were caught at the other five points, which were located in forested areas. In this reserve, five anopheline species were identified: *Anopheles aquasalis* (366/379), *An. mediopunctatus* (3/379), *An. shannoni* (1/379), *An. nuneztovari* (*s.l*.) (1/379) and *An.* (*Nyssorhynchus*) sp. (8/379) (Table 4). In Sítio Mangalho, within the Maracanã EPA, in the municipality of São Luís, 37 anophelines were caught (*An. aquasalis*, *An. goldii*, *An. evansae* and *Anopheles* sp.). In this place, mosquitoes were caught at three sampling points, between 4:00 pm and 10:00 pm. One point was located on the veranda of the house, for which protected human bait was used. Another sampling point, using a CDC-type light trap, was located in the farthest, most tree-covered area of the property, where non-human primates feed. The last sampling point was located at the extremity of the property, where there is a rustic swimming pool made of stones, which communicates with an arm of a river that runs through the property. In this area, a Shannon-type trap with light bait and protected human bait were used to catch mosquitos.

A considerable number of mosquitoes were caught on the veranda of the house, within only thirty minutes (9:30 pm - 10:00 pm), using protected human bait (16/37), all identified as *An. aquasalis*. The other mosquitoes were caught using a Shannon trap (in the forested area, using a light trap) (20/37), and one specimen was caught using protected human bait (1/37), at 4:30 pm on the first day of catching activities, while the area for installing the Shannon trap was being prepared (Table 4).

### Molecular identification of *Plasmodium* spp. and phylogenetic analysis

**Fig. 2:**
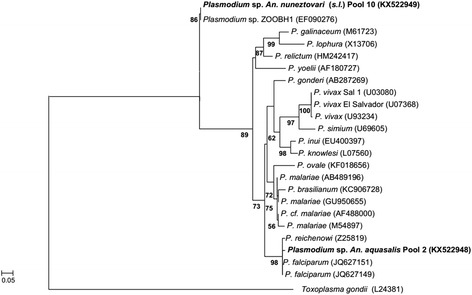
Phylogenetic relationships within the genus *Plasmodium* based on a 240 bp fragment of the 18S RNA gene. The phylogenetic tree was inferred using the maximum likelihood method and the GTR + G model. The sequences detected in the present study are highlighted in bold. Node numbers correspond to bootstrap values higher than 50% that were accessed with 1,000 pseudoreplicates. *Toxoplasma gondii* was used as the outgroup

Among the 54 pools of anophelines assayed using the protocol proposed by Santos et al. [15], two were positive for *Plasmodium*: pool 2, with ten specimens of *An. aquasalis* (Sítio Mangalho, municipality of São Luís); and pool 10, with one specimen of *An. nuneztovari* (*s.l*.) (Sítio Aguahy, municipality of São José de Ribamar). The amplified *Plasmodium* spp. sequences from the pools of *An. aquasalis* (pool 2) and *An. nuneztovari* (*s.l*.) (pool 10), respectively, were phylogenetically related to *P. falciparum* isolated from India (JQ627151, JQ627149), and *Plasmodium* sp. isolated from psittacines in Belo Horizonte Zoo (EF0902760) [26] (Fig. 2).

### Identification of the mosquito blood meal sources

Since protected human bait was used to catch mosquitos, the presence of human DNA in the pools was not considered to be a bias. Fragments of the cytochrome *b* gene of human DNA were identified in 44 (81.48%) out of the 54 pools that were assayed. This was already expected for *An. aquasalis*, since this species prefers human blood, the surveyed area has inhabitants which use the reserve for recreation and fishing.

DNA fragments of the cytochrome *b* gene of humans and dogs were identified in pool 4 of *An. aquasalis*. In pool 44, from the same species, only a fragment of cat *cytb* was identified. In 10 pools, no DNA fragments from the hosts that were assayed (humans, Neotropical primates, cats, dogs and birds) were amplified. The explanation for this finding may be that these anophelines had fed on another host whose DNA was not investigated or that they had not had any blood meal before the time when they were caught.

None of the samples amplified DNA fragments of the cytochrome *b* genes from birds or the β_2_-microglobulin of Neotropical primates.

## Discussion

Given the complexity of malaria vectors’ behaviour and the need to understand the epidemiological chain of the disease, the aim of the present study was to identify the blood meal sources of anopheline mosquitos from environmental preservation areas in which Neotropical primates are present, in two municipalities on São Luís Island, the State of Maranhão, Brazil. A total of six different species of anophelines were caught: *An. shannoni*, *An. mediopunctatus*, *An. goeldii*, *An. aquasalis*, *An. evansae* and *An. nuneztovari* (*s.l*.)*.* The last three of these species had already been identified in a previous study conducted in the municipality of Raposa, in the northernmost area of São Luís Island [27]. However, no previous study had reported the presence of the first three species mentioned above, on São Luís Island. Mosquitoes were caught over a 4-h period (5:30 pm - 9:30 pm) since a previous study in São Luis Island showed that *Anopheles* spp. presented higher biting activity from 6:00 pm - 9: 00 pm [3].

*Anopheles aquasalis* was the most frequent species in the areas sampled and accounted for 95.9% of the specimens collected. It was caught at both study sites, in São Luís and São José de Ribamar, in peridomestic and forested areas, respectively. In the state of Maranhão, this species presents high-frequency both in intra- and in peridomestic areas [28]. Rebêlo et al. [4] also reported a much higher number of this species in both of these municipalities, compared with other species of anophelines. This species is considered to be the main vector for human malaria on São Luís Island [28, 29] and it is an important vector in other areas of northern and northeastern Brazil. In other countries, such as Colombia [30], Venezuela [31] and French Guiana [32], *An. aquasalis* has been caught in high numbers and at various times of the day, even during the daytime [33]. This species of anophelines has natural exophilic and zoophilic behaviour [6, 34]. However, in cases of high density and absence of animals, it presents quite diverse feeding behaviour, e.g. feeding on human blood [6]. Therefore, some researchers have suggested that the classification of the feeding habits of this species should be changed from zoophilic to eclectic or opportunistic [5, 35].

In the present study, *An. aquasalis* showed the highest diversity of feeding sources. Among the 44 pools of this species, fragments of the human cytochrome *b* gene were identified in 37 pools. Moreover, in one pool, a fragment of dog DNA was also identified (Sítio Mangalho, Maracanã EPA, and municipality of São Luís). A fragment of domestic cat DNA was detected in another pool (Sítio Aguahy, municipality of São José de Ribamar). This corroborates the results found by Barros [36], who identified preferences for human (80%), bird and cat blood in specimens of *An. aquasalis* caught in the municipality of São José de Ribamar. Similar to the findings of Flores-Mendonza et al. [35], who identified a variety of blood meal sources used by this species of anopheline. Both of these authors used the precipitin test. The used anti-sera probably did not differentiate blood meals originating from humans and monkeys, and additional studies, more specific for the blood source, should be developed for this mosquito [37]. Therefore, because of the presence of Neotropical primates positive for *Plasmodium* [38], a PCR with specific primers for Neotropical primates was recommended. In the pools of *An. nuneztovari* (*s.l*.) (*n* = 1)*, An. shannoni* (*n* = 1)*, An. mediopunctatus* (*n* = 3)*, An. goeldii* (*n* = 1)*, An. evansae* (*n* = 1) and *An.* (*Nyssorhynchus*) sp. (*n* = 2) only fragments of human cytochrome *b* gene were identified. Preference among anophelines for dog and cat blood may be common in the areas studied here since those domestic animals were frequently present in both of these environmental conservation areas.

In 2013 (the period when these mosquitoes were collected), 48 cases of human malaria were reported in the municipality of São Luís in which the specimens of *An. aquasalis* were caught. Eight of these cases were due to *P. falciparum*, and 35 to *P. vivax* [2]. In turn, in the municipality of São José de Ribamar, the location where specimens of *An. nuneztovari* (*s.l*.) were caught, no cases of *P. falciparum* were reported in 2013 and 2014. This latter species is phylogenetically closer to the isolate of *Plasmodium* sp. that was found in wild birds in the state of Minas Gerais. In 2015, only one case of *P. falciparum* was reported in the municipality of São José de Ribamar [2].

Although no fragments of bird cytochrome *b* have been detected in *An. nuneztovari* (*s.l*.), the clade between the two isolates (*Plasmodium* sp. from mosquitoes and wild bird) is supported by a high bootstrap value (97). This anopheline species presents acrodendrophilic behaviour and is usually caught in higher numbers in the interior of the state of Maranhão [4]. It is usually more commonly found in forested areas than in human environments [6]. This species has already been reported by Deane et al. [39] in areas of the Brazilian Amazon region with the occurrence of simian malaria [40]. It has already been found naturally infected with *P. vivax* [6] and *P. falciparum* [41] in the Brazilian Amazon region and French Guiana, respectively. In Venezuela and Colombia, it is considered to be the primary malaria vector. In Brazil, in the state of Pará, specimens naturally infected with *Plasmodium* spp. have been reported [42].

Although *An. mediopunctatus, An. evansae* and *An. shannoni* were only found in low numbers in the present study, these species are important because they have already been identified in areas of occurrence of simian malaria [38, 39, 43–45]. These mosquitoes are more commonly found in treetops [46], but specimens were caught at ground level and using human bait in the present study, thus showing the versatility or opportunism of these vectors according to the offer of hosts. These species may be responsible for maintaining malaria in wild environments visited by humans and for human malaria outbreaks in unbalanced environments [6].

## Conclusions

*Anopheles aquasalis* was the most abundant species of anopheline in São Luís Island. *Plasmodium* spp. DNA was detected, thus confirming the importance of this species as the main vector on São Luís Island, Brazil. In addition, the presence of *An. nuneztovari* (*s.l*.) with DNA positive for *Plasmodium* spp. confirms its importance as a secondary vector. Occurrences of this species in forested areas where malaria cases have been reported need to be regarded as important. The sequencing results confirmed the presence of *Plasmodium* spp*.* in anophelines in the Sítio Aguahy Private Reserve, thus corroborating studies by our research group, which has already reported occurrences of this parasite in Neotropical primates.

## Abbreviations

EPA: Environmental Protection Area
FUNASA: National Healthcare Foundation
qPCR: Real time polymerase chain reaction
